# The Effect of Suvorexant on Fear Extinction Recall: A Double‐Blind Randomised Controlled Pilot Trial in Healthy Individuals

**DOI:** 10.1111/jsr.70033

**Published:** 2025-03-15

**Authors:** Maya T. Schenker, Lilith Z. Zeng, Joshua Lynskey, Matthew D. Greaves, Shima Rouhi, Amanda Kay, Andrew Dawson, Therese Thornton, Christian L. Nicholas, Kim L. Felmingham, Amy S. Jordan

**Affiliations:** ^1^ Melbourne School of Psychological Sciences The University of Melbourne Melbourne Australia

**Keywords:** DORA, extinction learning, fear acquisition, PTSD, Suvorexant, temazepam

## Abstract

Post‐traumatic stress disorder (PTSD) is a highly debilitating condition that develops after trauma exposure. Dysregulation in extinction memory consolidation (i.e., the ability to remember that trauma‐related stimuli no longer signal danger) is proposed to underlie PTSD development. Disruptions in rapid eye movement (REM) sleep are thought to be the key contributor to this dysregulation, as REM sleep is suggested to play a vital role in the processing of emotional memories. While previous literature has investigated the role of natural REM sleep variations or REM sleep disruptions on extinction recall capacities, none have attempted to increase REM sleep to improve extinction recall. In this pilot, randomised controlled trial, we investigated the effect of 20 mg suvorexant to increase REM sleep, 20 mg temazepam to decrease REM sleep, and a placebo on extinction recall in 30 healthy adults (age: *M* = 26.93 years, SD = 7.54). Overall, no difference in REM percentage (*p* = 0.68, *η* ^2^ = 0.0.03, small effect), nor in extinction recall (*p =* 0.58, *η* ^2^ = 0.04, small effect) was observed between the drug conditions. However, increased REM percentage was associated with decreased conditioned fear response at recall, indicating better extinction recall (*β* = −0.71, *p* = 0.03, *η*_p_^2^ = 0.10; moderate effect) across the sample. These findings suggest that increasing REM sleep in populations with REM disruptions such as PTSD to optimal levels could improve extinction recall. This underscores the potential of enhancing REM sleep as a therapeutic target for improving PTSD outcomes, warranting further investigation of suvorexant in clinical populations where REM sleep deficits are prevalent.

## 1 Introduction

Facing a traumatic event, such as assault, accident, natural disaster, or war, is very common, with an estimated 7 out of 10 people experiencing one or more traumatic events in their lives (Benjet et al. 2016). Following trauma exposure, post‐traumatic stress disorder (PTSD) develops if re‐experiencing symptoms (e.g., intrusions or nightmares), hyperarousal (e.g., sleeplessness), negative alterations in mood (e.g., depressive symptoms) and avoidance behaviour persist over time (American Psychiatric Association 2013). One of the proposed mechanisms underlying the development of PTSD is thought to involve impairments in fear extinction memory processing (Van Elzakker et al. 2014; Zuj et al. 2016). During the trauma, the individual learns to associate cues that are present during the traumatic event with the occurrence of the trauma and may show a conditioned fear response when encountering these cues outside the context of the trauma. After the trauma has passed, the conditioned response should gradually reduce over time through an extinction process, leading to recovery. However, in PTSD, extinction learning is thought to be impaired, leading to difficulty in extinguishing trauma‐related memories and poor adaptation to safe environments.

Extinction learning processes can be tested using an experimental Pavlovian fear conditioning and extinction learning paradigm. During fear acquisition, cues (conditioned stimuli or CS, e.g., coloured lights) presented on a computer screen are associated with an aversive outcome (e.g., electric shock). This is followed by the extinction learning phase where the CS is no longer paired with the aversive stimulus, resulting in the formation of a competing extinction memory trace, which inhibits the fear expression (Milad and Quirk 2012). After consolidating the fear and extinction memories, their respective strengths can be tested during extinction recall. Individuals with PTSD have been found to show a greater fear response, operationalised by increased psychophysiological arousal, during extinction recall than controls, reflecting impaired extinction recall (Milad et al. 2008, 2009; Pitman et al. 2012; Suarez‐Jimenez et al. 2020). Improving extinction capacities after trauma exposure can therefore be used to prevent PTSD development in the acute aftermath of trauma, enhance exposure therapy response, and prevent relapse from exposure therapy.

Sleep is important for the consolidation of memories, and ongoing disruption to sleep, particularly in rapid eye movement (REM) sleep, has been implicated as a catalyst for PTSD development after trauma exposure (e.g., Germain 2013; Pace‐Schott et al. 2015; Spoormaker and Montgomery 2008). REM sleep has been proposed to be the key sleep stage involved in emotional and fear memory consolidation (Walker and Stickgold 2004). For example, early disruptions in REM sleep following trauma exposure (i.e., shorter averaged REM segment lengths and lower REM beta frequencies) have been found to predict the development of PTSD symptoms (Mellman et al. 2007). Despite no overall difference in REM sleep, a recent meta‐analytic review reported that a subset of younger individuals with PTSD, where trauma exposure may be more recent, exhibited reduced REM sleep amount compared to healthy controls (Zhang et al. 2019). This underscores the potential importance of REM sleep, particularly in the early development of PTSD and its potential for targeted interventions.

Regarding fear conditioning, previous studies have reported mixed results for the role of REM sleep on extinction memory consolidation in both healthy controls and clinical samples. Our recent meta‐analysis (Schenker et al. 2021) found no overall association between REM sleep and extinction recall, including predominantly studies in healthy controls. It is noteworthy that all of the studies either assessed natural variations in REM sleep or compared experimentally disrupted REM sleep to normal sleep (Schenker et al. 2021). No previous study has attempted to increase or restore REM sleep, which may be a target for intervention to prevent PTSD development post‐trauma exposure.

Dual orexin receptor antagonists (DORAs), such as suvorexant, have been shown to increase REM sleep and may aid fear extinction learning following trauma exposure or in PTSD. The orexin system's main function is to modulate arousal. Suvorexant's soporific effect is based on blocking both orexin receptor 1 (OX1R) and 2 (OX2R), resulting in the dampening of wakefulness and thereby the promotion of sleep (Jacobson et al. 2014). In individuals with primary insomnia, suvorexant has been found to effectively increase total sleep time and REM sleep duration from the night after the first administration for up to 3 months of regular intake compared to placebo (Snyder et al. 2016). DORAs have also been found to decrease the latency of the first REM sleep episode without suppressing slow wave sleep (SWS, Snyder et al. 2016; Kuriyama and Tabata 2017). Two animal studies assessed the use of suvorexant to improve sleep‐dependent fear extinction consolidation with mixed findings. Clark et al. (2021) found increased REM sleep and enhanced extinction recall in mice treated with suvorexant, but no effect was found by Kobayashi and Forcelli (2024). Nonetheless, a translation of these findings to humans is warranted. If suvorexant successfully increases REM sleep and extinction memory consolidation in humans, suvorexant may be a viable option to support individuals suffering from trauma‐related sleep difficulties and thus reduce the chances of developing PTSD. This would provide an alternative to contraindicated drugs such as benzodiazepines, which are prescribed to up to 74% of individuals with PTSD (Guina et al. 2015), but have been found to disrupt mechanisms thought to underlie PTSD, including interfering with extinction learning (Makkar et al. 2010) and reducing REM sleep (de Mendonça et al. 2023; Schneider‐Helmert 1988).

The aim of the current study was to investigate the effect of suvorexant compared to a benzodiazepine (temazepam) and placebo on REM sleep and extinction recall in healthy adults. We hypothesised that (1) in the suvorexant group, REM sleep percentage would be increased compared to temazepam and placebo, (2) suvorexant would improve extinction recall compared to temazepam and placebo, and (3) improved extinction recall in the suvorexant group would be associated with increased REM percentage compared to both temazepam and placebo. Exploratory analyses assessed the association of other sleep stages, as well as sleep during the recovery night, on extinction recall. Finally, secondary analyses were conducted to assess whether there are safety‐related concerns related to suvorexant regarding next‐day sleepiness and vigilance (i.e., hangover effect). We hypothesised that there would be no difference between suvorexant and placebo on sleepiness and vigilance, but temazepam would be associated with greater sleepiness and lower vigilance.

## 2 Methods

### 2.1 Design

This study was a double‐blind randomised controlled trial investigating the effects of 20 mg suvorexant (Belsomra, Merck Sharp & Dohme, Australia), 20 mg temazepam (Temaze., Alphapharm, Australia) and placebo (sugar pill) on REM sleep and fear extinction recall (ACTRN12619001694101). For each participant, the study was conducted over approximately 2 weeks and included two in‐lab overnight stays to record polysomnography (PSG) and a two‐day fear conditioning and extinction learning paradigm. Recruitment commenced in 2019 but was put on hold from 2020 to 2022 due to COVID‐19‐related restrictions and laboratory closures. Data collection was resumed in 2023 and ended in 2024. The University of Melbourne (Australia) Human Research Ethics Committee approved this protocol (#130554).

### 2.2 Participants

**FIGURE 1:**
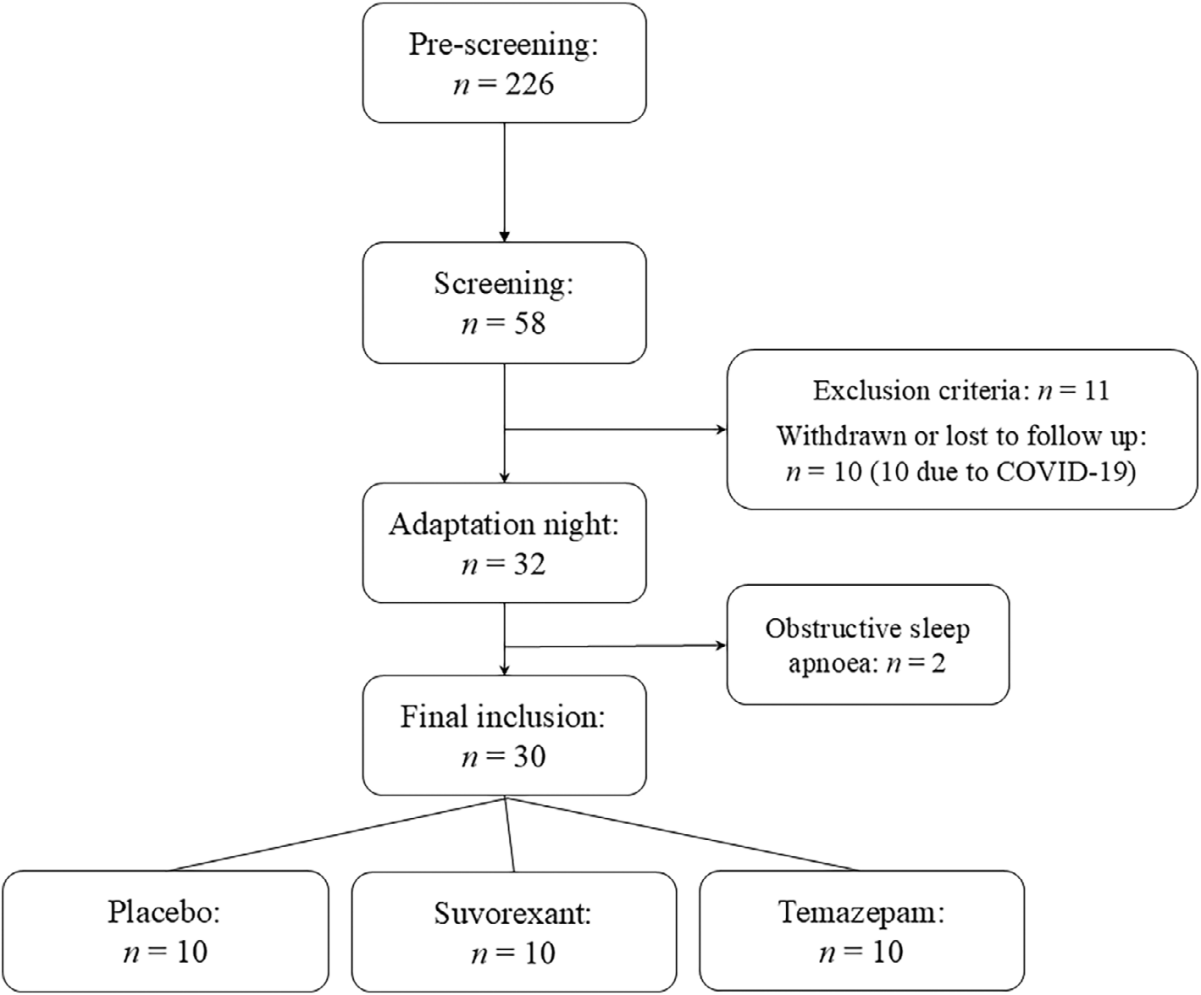
Participant recruitment flow chart.

Participants aged 18–55 were recruited through advertisements at the University of Melbourne. Exclusion criteria included the diagnosis of any current psychiatric disorder, and/or a total score above the threshold on the screening questionnaires (see below), physical disorders (e.g., severe hepatic or renal impairments), neurological disorders (e.g., narcolepsy, epilepsy, seizures), and/or cardiac disorders (e.g., hypotension or hypertension, i.e., blood pressure outside 90/60–140/90 mmHg). Further, unmanaged sleep disorders and sleep disturbances including jetlag or shift work, current medication intake that interacts with the study drugs and/or the central nervous system (e.g., sedatives, SSRIs), regular smoking, BMI less than 18.5 or greater than 30 kg/m^2^, and pregnancy, breastfeeding and/or trying to get pregnant were reasons for exclusion. Fifty‐six participants provided informed consent, and 30 participants completed the study. The recruitment process is shown in Figure 1. Due to delays resulting from the COVID‐19 lockdowns and limited funding and staffing available for study completion, the study was terminated before the a priori sample size was reached, and the study was reframed as a pilot project.

### 2.3 Procedure

Prospective participants completed an online pre‐screening survey to assess eligibility. Then, they were invited to a face‐to‐face screening in which blood pressure, height, and weight were measured, and they completed a battery of questionnaires. Eligible participants were scheduled for an adaptation night with full in‐lab PSG to account for the first night effect (Agnew Jr. et al. 1966) and to screen for sleep disorders, including obstructive sleep apnoea and restless leg syndrome. On the adaptation night, participants arrived 2 h before their habitual bedtime and stayed overnight. If no sleep disorder was detected, the test night was scheduled for about 1 week later, and participants were asked to maintain a regular sleep–wake schedule, refrain from napping during the day (monitored with a sleep diary and an actigraphy watch) and refrain from taking alcohol and drugs 48 h before the test night. On the test night, participants arrived 4 h before their habitual bedtime. They first completed a short questionnaire to assess compliance, were breathalysed (AcloSense Precision+, andatech, Nunawading, Australia) and biologically female participants provided a urinary sample for a pregnancy test (First Response, Church & Dwight Co. Inc., USA). They then completed the first three phases of the experimental paradigms (see below). PSG electrodes were attached, and 30 min before bedtime, participants received one of the study drugs (block‐randomised, stratified by sex). They were then given a 9‐h sleep opportunity with continuous PSG recording. After 9 h, participants were awoken, and PSG equipment was removed. Thirty minutes after wake‐up, they reported their level of sleepiness (see below) and underwent a 10‐min Vigilance Performance Test (PVT), and these were repeated four times every hour thereafter. After the first testing block, participants were offered breakfast and had the opportunity to shower. Between each block, participants were allowed to engage in activities of their choosing, but they were required to remain in the lab under the supervision of the investigators in case of any drug‐related adverse events. Five hours after wake‐up, participants were released with instructions not to drive or operate heavy machinery for the remainder of the day and to refrain from consuming alcohol, drugs, and grapefruit juice for 48 h. Participants then spent one night at home to allow for drug washout. During this recovery night, sleep was recorded at the participant's home to account for any sleep rebound effects before participants returned in the afternoon of the day after (~40 h after drug administration) to complete the second part of the experimental paradigm.

### 2.4 Materials

#### 2.4.1 Questionnaires

During the pre‐screening survey, participants responded to questions screening for exclusion criteria as well as basic demographic information (e.g., sex, age). If participants were deemed eligible, they then completed the Depression, Anxiety, and Stress Scale (DASS‐21, Lovibond and Lovibond 1995), the Trauma Exposure Questionnaire (TEQ, Vrana and Lauterbach 1994), the PTSD Checklist for DSM‐5 (PCL‐5, Weathers et al. 2013), the Pittsburgh Sleep Quality Index (PSQI, Buysse et al. 1989), the Epworth Sleepiness Scale (ESS, Johns 1991), and the Morningness Eveningness Questionnaire (MEQ, Horne and Östberg 1976). If participants scored moderate or higher on any DASS subscales (depression: ≥ 7, anxiety: ≥ 6, stress: ≥ 10, Lovibond and Lovibond 1995) and/or reached the clinical cut‐off on the PCL‐5 (≥ 33, Weathers et al. 2013), they were excluded, and referral options for psychological support were provided. On the morning after the test night, participants completed the Karolinska Sleepiness Scale (KSS, Åkerstedt and Gillberg 1990) hourly to assess potential drug‐related hangover effects. KSS is a 10‐point Likert scale: with 1 = *extremely alert*, 5 = *neither alert nor sleepy*, and 10 = *extremely sleepy, can't keep awake*.

#### 2.4.2 Fear Conditioning Paradigm

The fear conditioning and extinction learning paradigm was adapted from Milad et al. (2005). The CS consisted of a picture of either a red or blue desk lamp standing on a desk or shelf and was presented on a computer screen for 8000 ms. The unconditioned stimulus was a 500 Hz electric shock delivered for 500 ms. The paradigm consisted of four phases. During the habituation phase, participants familiarised themselves with the task by viewing two presentations of each CS colour without the shock. During the acquisition phase, one of the CS (CS+ or threat signal) was paired with the shock at CS offset using an 80% reinforcement rate, whereas the other CS was never reinforced (CS− or safety signal). A total of 10 trials was conducted (five each for CS+ and CS−). During the subsequent extinction phase, the context changed and consisted of two blocks of five trials each (early and late extinction). The final phase, extinction recall, was conducted at follow‐up 48 h later and was a repetition of either the early or late extinction phase. The colour‐context combinations were counterbalanced between participants and across drug conditions. Before the start of the experiment, electrodes to measure electrodermal activity (EDA) were attached to the intermediate phalanges of the participant's non‐dominant index and middle finger and a stimulator electrode was attached to the interosseous muscles of the other hand. The intensity of the electric shock was then calibrated to be highly uncomfortable, but below the individual's pain threshold. EDA, sampled at 512 Hz, was recorded using Labchart software and stored at 64 Hz (ADInstruments, Australia). To process the EDA data, it was first imported into MATLAB (version R2021b) and then converted to PsPM format (using PsPM toobox version 5.0.0, Bach 2024). Then, artefacts from movement were visually detected and interpolated with a linearly‐spaced vector using programs associated with Greaves et al. (2024). Finally, dynamic causal modelling (DCM) following Staib et al. (2015) was used to infer anticipatory sudomotor nerve activity from observed electrodermal activity separately for each individual within the 6‐s window after CS onset (Bach et al. 2010). We used the amplitude of the sudomotor nerve activity response, measured in sudomotor nerve units (1 unit corresponds to 1 μS peak change in skin conductance) for the analyses and will refer to this as skin conductance response (SCR).

#### 2.4.3 Sleep

Standard PSG according to AASM recommendations was performed using the Grael V2 PSG amplifier and Profusion PSG4 software (Compumedics Ltd., Abbotsford, Australia). For the recovery night, participants were either equipped with the Siesta ambulatory sleep system (data collected before the COVID‐19 pandemic) or Somfit (Compumedics Ltd., Abbotsford, Australia). Sleep stages were scored by a researcher blinded to the drug condition according to the AASM criteria (American Academy of Sleep Medicine 2023) for Siesta and Grael recordings or scored automatically for Somfit, with an algorithm validated to be comparable to manual PSG scoring (Miller et al. 2022). Actigraphy was measured using GENEActive Original wearables (Activinsights Ltd., Huntingdon, UK) and the Consensus Sleep Diary was used to monitor sleep subjectively (Carney et al. 2012).

#### 2.4.4 Vigilance Test

The PVT was conducted on a Windows laptop using a visual target. Lapses were defined as the number of reaction times > 500 ms within each trial, which is a commonly used measure of vigilance (Lim and Dinges 2008).

### 2.5 Statistical Analyses

To address Hypothesis 1, group differences in sleep between drug conditions during the test and recovery nights were calculated using one‐way analysis of variance (ANOVA). Data were square root transformed where the assumption of normality was not met as assessed by the Shapiro–Wilk test (Shapiro and Wilk 1965) and extreme outliers were winsorized to three times the interquartile range from the nearest quartile (Tabachnick and Fidell 2007). If the homoscedasticity assumption was violated or if square root transformation did not improve homoscedasticity, a Kruskal–Wallis test was used instead (Kruskal and Wallis 1952). Differences between test and recovery nights were analysed using a paired *t*‐test or Wilcoxon rank sum test where assumptions were violated. To test whether the experimental paradigm was successful, separate repeated measures ANOVAs were conducted for each phase using square root transformed SCR (SCRs) values. For the acquisition phase, a 2 (stimulus) by 4 (trial, excluding the first trial) ANOVA was used. Similarly, extinction learning was analysed using two separate 2 (stimulus) by 5 (trial) ANOVAs for the early (trials 1–5) and late phases (trials 6–10, Ney et al. 2021; Schenker et al. 2022; Zuj et al. 2018). For extinction recall, a paired *t*‐test was conducted to analyse differences between stimuli using the averaged SCRs during the first two trials (Schenker et al. 2022) and between drug conditions using an ANOVA (Hypothesis 2). Two separate linear models tested Hypothesis 3, including sleep, drug, and their interaction as predictors, and SCRs as outcome variables for each CS. Prior to running the models, assumptions were visually inspected, and extreme multivariate outliers were removed. Secondary analyses assessing next‐day sleepiness and vigilance were performed with One‐way ANOVAs to assess group differences between averaged KSS scores across trials as well as KSS per trial. Equivalent models were conducted for vigilance as measured by PVT lapses.

Post hoc multiple comparisons without a priori hypotheses were corrected using Benjamini and Hochberg (1995) procedure. All analyses were conducted in RStudio using R version 4.3.2 (2023).

A priori power analysis suggested a total sample size of 66 participants was sufficient to reach adequate power (1 − *β* = 0.80) based on the effect of suvorexant on REM sleep (*f* = 0.40, Snyder et al. 2016) and the effect of REM sleep on extinction recall (*f* ^2^ = 0.20, *α* = 0.05; Straus et al. 2017).

## 3 Results

Eighteen female and 12 male participants aged 26.93 years (± 7.54) completed the study. There were no significant differences between the groups in age, sex, or other baseline demographic variables (Table 1). The drugs were well tolerated, with only three mild adverse events reported. One participant who received suvorexant reported nausea in the morning, and two participants in the temazepam group reported light‐headedness and dizziness, respectively. Participants were more likely to guess that they received a placebo (65.38%, Table 2). During the test night, there were no significant group differences in any sleep stage or sleep stage percentages (Table 3). However, there was a significant difference in REM fragmentation or the number of spontaneous arousals per hour of REM sleep, χ^2^(2) = 6.67, *p* = 0.04, *η* ^2^ = 0.17 (large effect, Cohen 1988). Post hoc tests revealed significantly less REM fragmentation in the temazepam group compared to suvorexant (*p* = 0.04).

**TABLE 1:** Baseline demographics.

Measure	Total *M*	SD	Suvorexant *M*	SD	Temazepam *M*	SD	Placebo *M*	SD	*p*	*η* ^2^
Age	26.93	7.54	25.00	6.11	27.90	8.99	27.90	7.68	0.54	0.03
PSQI	3.97	1.79	4.60	1.78	4.10	1.60	3.20	1.87	0.19	0.05
MEQ	39.07	7.53	40.90	7.14	34.90	6.45	41.40	7.81	0.09^a^	0.16
ESS	4.80	3.66	5.70	3.74	4.20	3.36	4.5	4.06	0.55^a^	0.04
PCL‐5	5.17	6.95	8.70	9.81	3.10	2.92	3.70	5.48	0.22	0.04
DASS‐D	7.20	2.09	7.10	2.03	7.40	2.12	7.10	2.33	0.90	0.07
DASS‐A	6.90	3.17	6.90	2.89	7.90	4.10	5.90	2.23	0.30	0.01
DASS‐S	2033	2.31	3.00	1.89	3.10	3.00	1.80	1.87	0.34	0.01

*Note: n* = 30. The test statistic is based on Kruskal–Wallis due to nonnormality unless otherwise specified. η^2^ ≈ 0.01 (small effect), η^2^ ≈ 0.06 (moderate effect), η^2^ ≈ 0.14 (large effect).

Abbreviations: DASS = Depression, Anxiety and Stress Scale (21‐item), ESS = Epworth Sleepiness Scale, MEQ = Morning Eveningness Questionnaire, PCL‐5 = PTSD Checklist for DSM‐5, PSQI = Pittsburgh Sleep Quality Index.

^a^ One‐way ANOVA.

**TABLE 2:** Perceived treatment condition and guess accuracy.

Guess	Placebo	Suvorexant	Temazepam	Total
Correct	6	4	4	14
Incorrect	1	6	5	12
Total	7	10	9	26

*Note*: The guess in the suvorexant and temazepam condition was not specific to the drug. 17 guessed placebo and 9 guessed drug.

**TABLE 3:** Group difference in sleep during test night (one‐way ANOVA).

Measure	Total *M*	SD	Suvorexant *M*	SD	Temazepam *M*	SD	Placebo *M*	SD	*p*	*η* ^2^
Total sleep time	481.40	28.89	491.75	30.62	479.80	31.53	472.65	23.46	0.39	0.08
Sleep onset latency	14.28	10.77	11.550	5.53	19.30	14.17	12.00	10.01	0.41^a^	0.01
Wake after sleep onset	25.10	14.72	16.95	7.45	27.20	12.41	31.15	19.25	0.07^b^	0.18
Sleep efficiency (%)	92.23	3.39	94.00	2.40	91.10	3.51	91.60	3.69	0.11^c^	0.15
*N*_1_ minutes	27.82	11.90	31.55	14.49	21.45	9.35	30.45	9.53	0.13^b^	0.16
*N*_2_ minutes	242.75	31.98	243.95	31.37	252.50	26.96	231.80	36.66	0.36	0.07
SWS minutes	87.67	31.99	85.65	27.95	85.00	35.59	93.25	34.67	0.83	0.01
REM minutes	122.18	24.83	130.60	29.51	118.80	19.67	117.15	23.20	0.42	0.06
REM latency	90.10	42.91	91.40	55.07	100.50	44.99	78.40	24.51	0.58^b^	0.04
REM fragmentation^d^	16.33	6.29	18.60	4.99	12.50	3.27	17.90	8.20	0.04* ^,^ ^a^	0.17
*N*_1_ (%)	5.80	2.43	6.41	2.88	4.57	2.14	6.43	1.89	0.14	0.13
*N*_2_ (%)	50.51	5.96	49.61	5.29	52.88	4.66	49.05	7.42	0.31	0.08
SWS (%)	18.30	6.38	17.54	5.79	17.61	6.70	19.74	7.03	0.69	0.03
REM %)	25.40	4.61	26.48	5.18	24.95	4.00	24.78	4.88	0.68	0.03

*Note: n* = 30. Total sleep time, sleep onset latency, wake after sleep onset, and REM latency in minutes. Sleep efficiency: time asleep during sleep opportunity. η^2^ ≈ 0.01 (small effect), η^2^ ≈ 0.06 (moderate effect), η^2^ ≈ 0.14 (large effect).

Abbreviations: *N*_1_ = NREM Stage 1, *N*_2_ = NREM stage 2, REM = rapid eye movement sleep, SWS = Slow wave sleep.

^a^ Kruskal–Wallis test statistic due to assumption violation.

^b^ Test statistic based on sqrt transformed data.

^c^ Test statistic based on winsorized data.

^d^ Number of spontaneous arousals per hour of REM sleep.

* *p* < 0.05.

### 3.1 Fear Conditioning Paradigm

**FIGURE 2:**
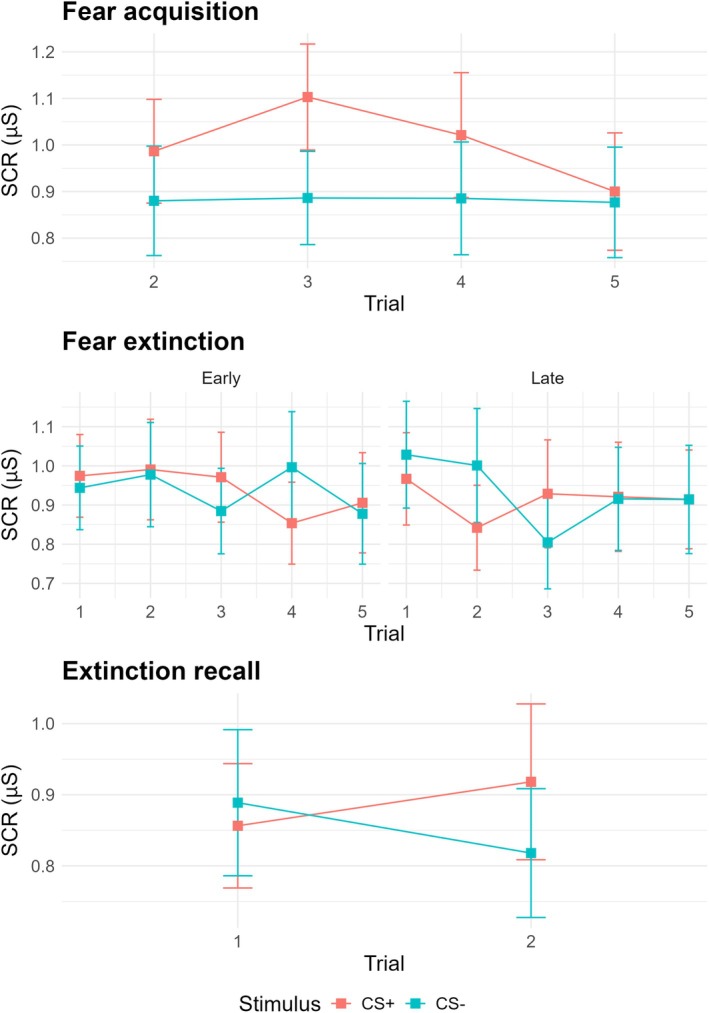
Fear acquisition and extinction learning paradigm. *Note*: Error bars depict standard error.

During the fear acquisition phase, there was a significant main effect for stimulus, *F*(1,29) = 6.99, *p* = 0.01, *η* ^2^ = 0.19 (large effect). SCR was significantly larger for the CS+ than the CS−, *t*(119) = 3.23, *p* = 0.001, *d* = 0.30 (small effect, see Figure 2, top), indicating successful fear acquisition. There was no longer a significant difference between the CS+ and CS− during the early, *F*(1,29) = 0.01, *p* = 0.94, *η* ^2^ < 0.01 (negligible effect), and late extinction phases, *F*(1,29) = 0.17, *p* = 0.68, *η* ^2^ = 0.01 (negligible effect, see Figure 2, middle), indicating that extinction learning had occurred. At recall, a paired *t*‐test showed that extinction learning was maintained, with no significant difference in SCR between stimuli, *t*(29) = 0.49, *p* = 0.63, *d* = 0.09 (negligible effect, see Figure 2, bottom).

### 3.2 Extinction Recall

At recall, there was no significant difference in SCR between the drug conditions in extinction recall (CS+), *F*(2,27) = 0.13, *p* = 0.58, *η* ^2^ = 0.04 (small effect), nor in safety recall (CS−), *F*(2,27) = 0.35, *p = 0*.50, *η* ^2^ = 0.05 (small effect). Despite these non‐significant differences, we assessed the combined effect of REM percentage and drug condition on extinction (CS+) and safety recall (CS−).

**FIGURE 3:**
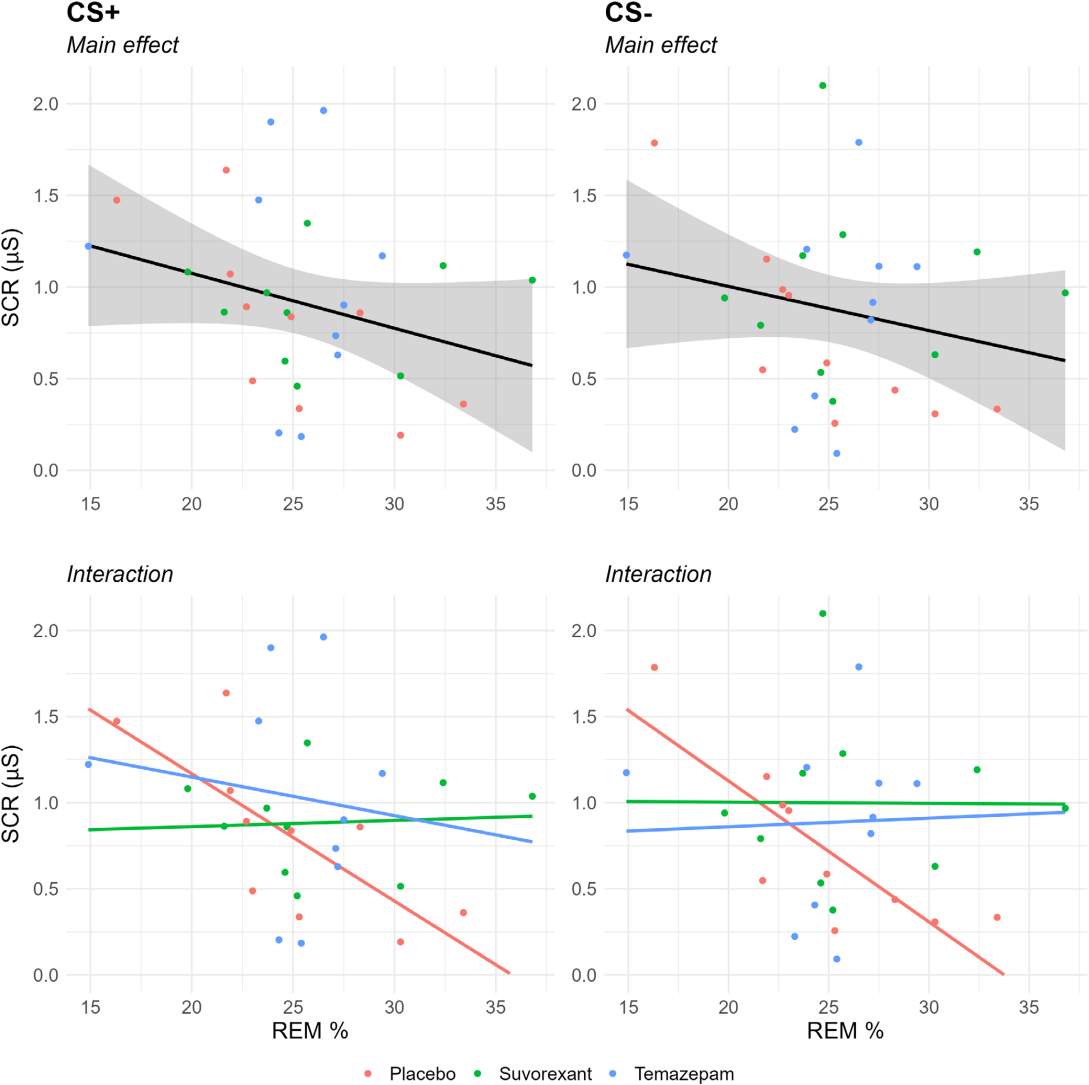
Association between REM percentage, drug condition, and extinction recall. *Note*: Error bands depict standard error.

For the CS+, there was a significant main effect for REM percentage (*p* = 0.03, *η*_*p*_^2^ = 0.10; moderate effect, centred due to concerns of multicollinearity), but not for drug condition (*p* = 0.47, *η*_*p*_^2^ = 0.05; small effect), nor the REM percentage by drug interaction (*p* = 0.22, *η*_*p*_^2^ = 0.12; moderate‐large effect). Increased REM percentage was associated with a reduction in SCR, suggesting better extinction recall (*β* = −0.71, 95% CI [−0.14, −0.01]). To assess the association between REM percentage and extinction recall in each drug condition, separate linear regressions were conducted post hoc. In the placebo group, increased REM percentage was associated with a decrease in SCR at recall (*β* = −0.71, 95% CI [−0.13, −0.02], *p* = 0.01, *η*_*p*_^2^ = 0.56; large effect), indicating better extinction recall. Conversely, neither suvorexant (*β* = 0.06, 95% CI [−0.04, 0.05], *p* = 0.86, *η*_*p*_^2^ = 0.00) nor temazepam (*β* = −0.14, 95% CI [−0.15, 0.10], *p* = 0.69, *η*_*p*_^2^ = 0.02) was associated with SCR at recall (see Figure 3), left side.

Similarly for the CS−, there was a significant main effect for REM percentage (*β* = −0.77, 95% CI [−0.15, −0.02], *p* = 0.02, *η*_*p*_^2^ = 0.06; moderate effect), but not for drug condition (*p* = 0.34, *η*_*p*_^2^ = 0.08; moderate effect), nor the REM percentage by drug interaction (*p* = 0.13 *η*_*p*_^2^ = 0.18; large effect). Post hoc tests again showed a significant association in the placebo group between REM percentage and safety recall (*β* = −0.83, 95% CI [−0.13, −0.04], *p* = 0.003, *η* ^2^ = 0.68; large effect), but not the suvorexant (*β* = −0.01, 95% CI [−0.08, 0.08], *p* = 0.98, *η* ^2^ < 0.01, negligible effect), nor temazepam groups (*β* = 0.04, 95% CI [−0.10, 0.11], *p* = 0.92, *η* ^2^ < 0.01, negligible effect) (see Figure 3, right side). The results regarding the other sleep stages and sleep parameters are included in Table S1A (extinction recall) and B (safety recall).

### 3.3 Recovery Night

During the recovery night, the sleep of six participants was measured using Siesta, and the remaining wore Somfit. Data from seven participants were missing (three placebo, two suvorexant, three temazepam). Compared to the test night, participants had significantly less total sleep time, *t*(22) = 6.18, *p* < 0.001, *d* = 1.29 (large effect), and significantly lower sleep efficiency, *W* = 211.5, *z* = 8.00, *p* = 0.006, *r* = 0.58 (moderate‐large effect). Consequently, there was significantly less *N*_1_, *N*_2_, and REM minutes during the recovery night (*p*'s < 0.05), but there were no differences in sleep stage percentages between nights (Table S2). During the recovery night, there were no statistically significant differences in sleep parameters between groups (see Table S3). With regards to the association between REM percentage during the recovery night and extinction or safety recall, exploratory analyses showed no significant effect nor interaction with the drug condition (*p*'s > 0.05).

### 3.4 Hangover Effect

**FIGURE 4:**
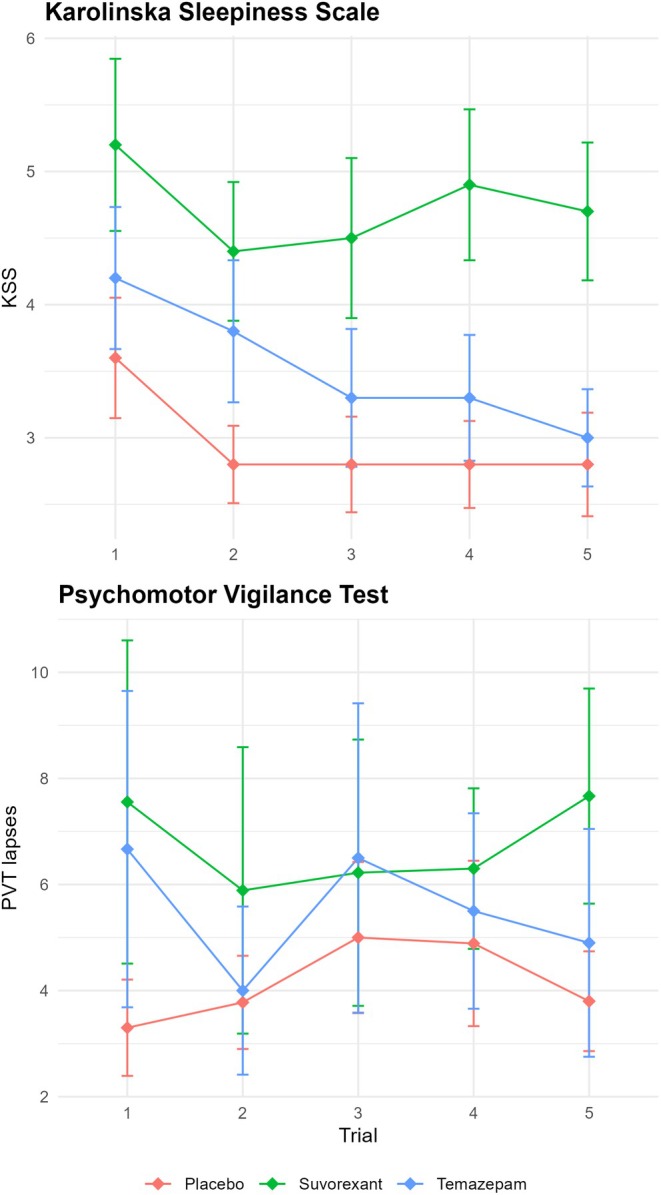
Karolinska Sleepiness Scale (KSS) and Psychomotor Vigilance Test (PVT). *Note*: Error bars depict standard error.

Average scores on the morning after the test night, participants reported feeling rather alert (*M* = 3.92, SD = 1.46). Overall, mean scores were less than five across all trials per group (except for the first trial in the suvorexant group, *M* = 5.20, see Figure 4), indicating that participants felt more alert than sleepy. However, mean sleepiness scores were significantly different between groups, *F*(2,27) = 5.02, *p* = 0.01, *η* ^2^ = 0.27 (large effect) with those in the suvorexant group reporting feeling less alert than the temazepam (*p* = 0.04) and placebo (*p* = 0.02) groups. While there was no statistical group difference for earlier trials, there were significant differences for Trials 4, *F*(2,27) = 4.79, *p* = 0.02, *η* ^2^ = 0.26 (large effect), and 5, *F*(2,27) = 5.92, *p* = 0.007, *η* ^2^ = 0.30 (large effect). In both these trials, those who received suvorexant reported feeling less alert than both those who received temazepam (Trial 4, *p* = 0.03; Trial 5, *p* = 0.01) and those who received placebo (Trial 4, *p* = 0.01; Trial 5, *p* = 0.01). A similar pattern of results was seen for PVT lapses but did not reach statistical significance (Figure 4). It should be highlighted that for the PVT, there were missing data for 12 trials (8%), five in the temazepam group, four in the suvorexant group, and three in the placebo group. Test statistics are included in Tables S4–S7.

## 4 Discussion

The aim of this study was to investigate the effect of suvorexant on REM sleep and fear extinction recall in healthy individuals. The smaller‐than‐planned sample size and resulting reduced power mean that the results from this study must be interpreted with caution. Nonetheless, this study provides preliminary insight into directional trends of the potential of psychopharmacological manipulations of REM sleep as a means to modify extinction recall.

Contrary to our first hypothesis, no difference in REM percentage was found in a single dose of 20 mg of suvorexant compared to a single dose of 20 mg temazepam or placebo. Given that the participants in this study were healthy individuals without sleep disorders, this may not be surprising. A recent systematic review published after the start of this trial reported that suvorexant may not affect REM sleep amounts in healthy controls (Clark et al. 2020). Also contrary to our expectations, temazepam did not result in the expected REM sleep suppression effect in our study. Again, temazepam's REM‐suppressive effects may be less pronounced in healthy individuals (e.g., Feinberg et al. 2000; Hemmeter et al. 2000) compared to clinical populations (e.g., Schneider‐Helmert 1988; Vgontzas et al. 1994). Therefore, these results do not rule out the possibility that suvorexant may have a REM‐enhancing effect in clinical populations with REM disruptions, such as PTSD.

The second hypothesis was also not supported as no difference in extinction or safety recall was observed between drugs. Beyond the modulation of arousal and wakefulness, the orexin system, which suvorexant targets, is further involved in the processing of fear and emotional responses (Soya and Sakurai 2020). Particularly, blocking OX1R has been beneficial in reducing fear acquisition, improving extinction learning, and reducing fear recall in animal models (Flores et al. 2017, 2014; Soya et al. 2017). Although we did not observe any direct effect of suvorexant on extinction recall in this study, the question is still open whether orexin antagonists may enhance extinction learning and recall in PTSD, processes that are commonly impaired in these individuals (e.g., Suarez‐Jimenez et al. 2020).

Regardless of the non‐significant drug effects on REM sleep and extinction recall, we found partial support for our third hypothesis. Although suvorexant was not associated with increased REM sleep or improved extinction consolidation, there was a significant negative association between REM percentage across all groups and SCR during the first two trials at extinction recall, supporting the idea that increased REM sleep promoted extinction recall. This is consistent with previous research reporting that more REM sleep is associated with improved extinction recall (e.g., Menz et al. 2013; Pace‐Schott et al. 2014; Spoormaker et al. 2012, 2010). A similar result was found for safety recall. In line with previous research, increased REM sleep was associated with lower SCR at recall indicating better safety retention (Lerner et al. 2021; Menz et al. 2016; Straus et al. 2018). Interestingly, in our study, this effect may have been driven by the placebo group, with no significant association in either drug condition. This suggests that extinction recall may depend on the optimal amount of REM sleep that occurs naturally. However, since suvorexant can restore REM sleep in clinical samples to levels seen in healthy individuals (Snyder et al. 2016), it remains plausible that suvorexant may improve extinction recall in individuals with PTSD through increased REM sleep disruptions. This question warrants further exploration in adequately powered studies, as the lack of effect of suvorexant on the REM‐extinction recall relationship may be attributable to the small sample size and reduced power to detect an effect.

Looking at the safety aspect of the drugs, there were no notable hangover effects of suvorexant or temazepam during the morning following the test night. Even though the participants who received suvorexant scored significantly higher on the KSS, they did not report feeling sleepy (i.e., KSS > 5). While it may be expected that individuals without sleep issues who received a sleeping drug might feel sleepier in the morning than those who received a placebo, interestingly, subjective sleepiness in the morning did not differ between the temazepam condition and the placebo group. This can perhaps be attributed to the more rapid elimination of temazepam, which has a half‐life as low as 8 h (Bittencourt et al. 1979; Fuccella 1979), compared to suvorexant, which has a substantially larger half‐life of approximately 12 h (Australian Product Information 2018). At the time of the last trial, 14 h after drug ingestion, individuals who received suvorexant would still have had approximately 45% of the substance remaining in their body, whereas 70% of the temazepam would have been eliminated by that time. This may explain why those who received suvorexant felt less alert than those who received temazepam and, if given the opportunity, may have slept longer. Despite significant differences in the total amount of sleep between the test and recovery nights, there was no difference in the total amount of sleep between groups. This suggests that the individuals who received suvorexant had adequate amounts of sleep during the test night but could have slept longer because they were more sedated.

Results for vigilance, as measured by lapses in reaction time in the PVT, conformed to expected outcomes. The number of lapses that occurred in this study was generally higher than those found in previous research after a full night of sleep in healthy individuals (e.g., Jongen et al. 2015; McMahon et al. 2018), which could be attributed to our participants sleeping in a lab environment with PSG equipment. Consistent with a previous study comparing lapses in vigilance between placebo and suvorexant groups (Caldwell et al. 2022), we found no impairment in vigilance following one night of suvorexant. Taken together, the findings from this study suggest that there should be little concern regarding substantial hangover effects after one night of suvorexant, given enough sleep opportunity.

### 4.1 Limitations and Future Directions

There were several limitations to this study. First and foremost, the small number of participants resulted in low power. Replication of our results in a larger sample will be required to confirm whether the observed effects are true.

Secondly, in this study, the fear conditioning and extinction learning paradigm took place in the evening about 4 h before bedtime. Previous studies have found a strong circadian influence on the capacity to acquire fear and extinction memories. For example, Pace‐Schott et al. (2013) found a notable time‐of‐day effect with superior extinction recall if extinction was learned in the morning. Glucocorticoids, including cortisol, are known to facilitate extinction memory consolidation (de Quervain et al. 2017) and are naturally elevated in the morning (Bailey and Heitkemper 2001). Therefore, the effect found in this study might be dampened due to the timing of the experiment.

Another factor that might have impacted our results was the dosage of the study drugs, which was not adjusted for participants' height, weight or sex. In female individuals and those with higher body fat, the maximum serum concentration of suvorexant per dose may be higher, with slower absorption and clearance rates (Australian Product Information 2018). Similar sex differences have been found for temazepam (Divoll et al. 1981). Despite equal numbers of female and male participants in our study and the exclusion of obese participants, sex and body fat are confounding variables that could be controlled for in future research, or individualised dosage adjustments could be made.

Finally, since the start of this study, other dual orexin receptor antagonists have been approved. For example, lemborexant is approved in multiple countries, including Australia and the US. Lemborexant has the same mechanism of action but a higher affinity to the orexin receptor 2 than suvorexant (Kishi et al. 2020). While there is not yet enough research to suggest that lemborexant is safe or effective in individuals with PTSD, this could be considered in future research.

### 4.2 Clinical Implications

The purpose of this study was to investigate the effect of suvorexant on the mechanisms underlying sleep‐dependent fear conditioning and extinction learning processes in healthy controls, which is relevant to PTSD. While this study found no effect of suvorexant on REM sleep and fear extinction recall, the reduced power may have limited the ability to detect a drug effect. These findings in healthy individuals do not preclude the possibility that this effect is present in individuals with PTSD. PTSD is commonly characterised by disruptions in REM sleep (Zhang et al. 2019) and impairments in extinction learning and consolidation (e.g., Guthrie and Bryant 2006; Zuj and Norrholm 2019), neither of which were observed in our sample of healthy individuals with normal sleep. In addition, our meta‐analysis found preliminary evidence that the relationship between REM sleep and extinction recall may differ between clinical populations and controls (Schenker et al. 2021). Therefore, an appropriately powered replication of our study in a clinical sample with PTSD is important.

Recent studies have found that suvorexant is safe and effective in PTSD, and that in this population it increases REM percentage from baseline (Mellman et al. 2022), increases REM segment duration, and decreases REM latency compared to placebo (Kobayashi and Forcelli 2024; Mellman et al. 2022). In addition, suvorexant has been found to be associated with a reduction in PTSD symptom severity (however unspecific to the drug, Kobayashi and Forcelli 2024; Mellman et al. 2022), and to promote between‐session habituation in an exposure‐based treatment (Kobayashi and Forcelli 2024). While no study has yet investigated the effect of suvorexant or other dual orexin receptor antagonists on sleep in individuals in the immediate aftermath of a traumatic event, such drugs might hold potential for promoting adaptive, sleep‐dependent memory consolidation, which could ultimately help to reduce the risk of developing PTSD.

### 4.3 Conclusion

This double‐blind, placebo‐controlled pilot study assessing the effect of suvorexant compared to temazepam and placebo on REM sleep and fear extinction recall found no evidence of group differences in REM sleep and no effect of suvorexant on extinction recall. There was preliminary evidence, however, that greater REM percentage across groups was associated with lower SCR, indicative of better extinction recall. Even though this effect may have been driven by the placebo group, the potential remains that in individuals with PTSD with disrupted sleep, suvorexant may restore REM sleep and therefore facilitate extinction recall. Despite the limitations of this study, suvorexant may support the consolidation of extinction memories in individuals who have experienced a traumatic event.

## Author Contributions

**Maya T. Schenker:** conceptualization, investigation, writing – original draft, methodology, visualization, formal analysis, data curation. **Lilith Z. Zeng:** investigation, writing – review and editing. **Joshua Lynskey:** investigation, writing – review and editing. **Matthew D. Greaves:** writing – review and editing, software. **Shima Rouhi:** investigation, writing – review and editing. **Amanda Kay:** investigation, writing – review and editing. **Andrew Dawson:** writing – review and editing, formal analysis. **Therese Thornton:** methodology, writing – review and editing. **Christian L. Nicholas:** supervision, writing – review and editing. **Kim L. Felmingham:** writing – review and editing, resources, supervision. **Amy S. Jordan:** writing – review and editing, resources, supervision.

## Conflicts of Interest

The authors declare no conflicts of interest.

## Supporting information


**Data S1.** Supporting Information.

## Data Availability

The data that support the findings of this study are available on request from the corresponding author. The data are not publicly available due to privacy or ethical restrictions.
