# Supplementary material for: The Effect of Suvorexant on Fear Extinction Recall: A Double‐Blind Randomised Controlled Pilot Trial in Healthy Individuals
Source: J Sleep Res. 2025 Mar 15;34(6):e70033. doi: 10.1111/jsr.70033 (PMC12592838; doi:10.1111/jsr.70033)
Supplement: Supplementary file 1 — Data S1. Supporting Information. [file JSR-34-e70033-s001.docx]

The effect of suvorexant on fear extinction recall: a double-blind randomised controlled pilot trial in healthy individuals - supplement

| Table S1A: Model results including other sleep stages and drug effect on extinction recall (CS+). | | | | | | | | | | | | | | | |
| --- | --- | --- | --- | --- | --- | --- | --- | --- | --- | --- | --- | --- | --- | --- | --- |
|  | N1 % | | N2% | | SWS % | | REM latency | | TST | | WASO | | SOL | |  |
|  | *p* | *η_p_^2^* | *p* | *η_p_^2^* | *p* | *η_p_^2^* | *p* | *η_p_^2^* | *p* | *η_p_^2^* | *p* | *η_p_^2^* | *p* | *η_p_^2^* |  |
| Sleep effect | .74 | 0.00 | .10 | **0.27** | .85 | 0.06 | .21 | **0.14** | .57 | 0.00 | .10 | **0.19** | .08 | 0.06 |  |
| Drug effect | .60 | 0.04 | .97 | 0.00 | .65 | 0.03 | .90 | .02 | .55 | 0.04 | .50 | **0.10** | .57 | 0.04 |  |
| Interaction | .89 | 0.00 | .71 | 0.03 | .80 | 0.02 | .51 | .05 | .77 | 0.02 | .31 | **0.09** | .24 | **0.12** |  |
| *Note:* TST: Total sleep time, WASO: Wake after sleep onset, SOL: sleep onset latency.  η_p_^2^ ≈ 0.01 (small effect), η_p_^2^ ≈ 0.06 (**moderate effect**), η_p_^2^ ≈ 0.14 (**large effect**)  * *p* <.05 | | | | | | | | | | | | | | | |

| Table S1B: Model results including other sleep stages and drug effect on safety recall (CS-). | | | | | | | | | | | | | | | |
| --- | --- | --- | --- | --- | --- | --- | --- | --- | --- | --- | --- | --- | --- | --- | --- |
|  | N1 % | | N2% | | SWS % | | REM latency | | TST | | WASO | | SOL | |  |
|  | *p* | *η_p_^2^* | *p* | *η_p_^2^* | *p* | *η_p_^2^* | *p* | *η_p_^2^* | *p* | *η_p_^2^* | *p* | *η_p_^2^* | *p* | *η_p_^2^* |  |
| Sleep effect | .09 | 0.05 | .94 | 0.04 | .71 | 0.00 | .14 | **0.09** | .36 | **0.10** | .01* | **0.12** | .76 | 0.03 |  |
| Drug effect | .43 | 0.02 | .24 | **0.11** | .55 | 0.05 | .68 | 0.05 | .37 | **0.90** | .40 | **0.11** | .51 | **0.07** |  |
| Interaction | .04* | **0.25** | .81 | 0.02 | .70 | 0.03 | .20 | **0.13** | .86 | 0.01 | .19 | **0.14** | .56 | 0.05 |  |
| *Note:* TST: Total sleep time, WASO: Wake after sleep onset, SOL: sleep onset latency.  η_p_^2^ ≈ 0.01 (small effect), η_p_^2^ ≈ 0.06 (**moderate effect**), η_p_^2^ ≈ 0.14 (**large effect**)  * *p* <.05 | | | | | | | | | | | | | | | |

| Table S2: Difference between test and recovery night (paired t-test) | | | | | |
| --- | --- | --- | --- | --- | --- |
| Sleep | Difference *M (SD)* | *t* | *df* | *p* | *d* |
| Total sleep time | 109.80 (90.91) | 5.79 | 22 | <.001*** | **1.21** |
| Sleep onset latency | 3.10 (15.93) | 0.97 | 24 | .34 | 0.19 |
| Wake after sleep onset | -29.13 (64.05) | -2.45 | 22 | .02* ^a^ | -**0.51** |
| Sleep efficiency % | 8.98 (13.55) | 213.5 |  | .006* ^c^ | **0.64** |
| N1 minutes | 11.80 (15.13) | 3.74 | 22 | .001** | **0.78** |
| N2 minutes | 58.13 (63.30) | 4.40 | 22 | <.001*** | 0.28 |
| SWS minutes | 9.28 (41.59) | 1.35 | 22 | .19 | 0.28 |
| REM minutes | 29.26 (41.59) | 3.37 | 22 | .003** | **0.70** |
| REM latency | 16.88 (51.39) | 1.61 | 23 | .12 | 0.33 |
| N1 % | 1.81 (3.36) | 2.58 | 22 | .02* | **0.54** |
| N2 % | 1.5 (8.39) | 0.87 | 22 | .39 | 0.18 |
| SWS % | -2.94 (8.53) | -1.65 | 22 | .11 | -0.34 |
| REM % | 0.97 (7.58) | 0.62 | 22 | .54 | 0.13 |
| *Notes*: n test night =30, n recovery night = 23 (25 for sleep onset latency, 24 for REM latency). df: degrees of freedom.  d =0.20 (small effect), d = 0.50 (**moderate effect**), d = 0.80 (**large effect**)  ** p* ≤ .05, *** p* ≤ .01, **** p* ≤ .001  ^a^ Wilcoxon rank sum test due to assumption violation, ^b^ Test statistic based on sqrt transformed data | | | | | |

| Table S3: Group differences during recovery night (one-way ANOVA) | | | | | | | | | | |
| --- | --- | --- | --- | --- | --- | --- | --- | --- | --- | --- |
| **Measure** | **Total *M*** | ***SD*** | **Suvorexant *M*** | ***SD*** | **Temazepam *M*** | ***SD*** | **Placebo *M*** | ***SD*** | ***p*** | ***η^2^*** |
| Total sleep time | 371.17 | 76.55 | 345.44 | 89.97 | 362.06 | 66.27 | 411.00 | 63.82 | .24 | **0.13** |
| Sleep onset latency | 11.50 | 16.86 | 11.44 | 18.31 | 12.6 | 21.70 | 10.00 | 5.89 | .33^a^ | 0.01 |
| Wake after sleep onset | 53.00 | 64.48 | 58.88 | 87.29 | 60.94 | 68.17 | 37.21 | 22.58 | .86^a^ | 0.06 |
| Sleep efficiency % | 83.41 | 13.07 | 77.03 | 19.08 | 86.03 | 8.09 | 87.73 | 6.28 | .56^a^ | 0.03 |
| N1 minutes | 16.09 | 9.56 | 11.81 | 8.03 | 18.94 | 11.74 | 17.71 | 7.78 | .28^c^ | **0.12** |
| N2 minutes | 182.50 | 47.50 | 175.19 | 53.53 | 175.31 | 47.44 | 199.07 | 42.94 | .56 | 0.06 |
| SWS minutes | 79.39 | 36.99 | 81.69 | 30.69 | 67.06 | 40.40 | 90.86 | 40.62 | .47 | 0.07 |
| REM minutes | 93.63 | 34.72 | 77.38 | 31.70 | 101.13 | 33.69 | 103.64 | 37.15 | .27 | **0.12** |
| REM latency | 79.42 | 35.56 | 89.06 | 47.02 | 70.50 | 34.40 | 79.86 | 21.11 | .55^b^ | 0.05 |
| N1 % | 4.02 | 2.56 | 3.28 | 2.66 | 4.84 | 2.84 | 3.93 | 2.16 | .44^c^ | 0.08 |
| N2 % | 48.63 | 7.55 | 50.26 | 7.47 | 47.28 | 8.07 | 48.31 | 7.87 | .74 | 0.03 |
| SWS % | 21.369 | 9.11 | 23.13 | 4.99 | 18.89 | 11.96 | 22.16 | 9.78 | .81 | 0.06 |
| REM % | 24.65 | 7.04 | 21.96 | 6.75 | 27.36 | 6.83 | 24.61 | 7.39 | .15 | 0.07 |
| *Notes*: n=23. Total sleep time, sleep onset latency, wake after sleep onset and REM latency in minutes. Sleep efficiency: time asleep during sleep opportunity. N1: NREM stage 1, N2: NREM stage 2, SWS: Slow wave sleep, REM: Rapid eye movement sleep.  η*^2^* ≈ 0.01 (small effect), η*^2^* ≈ 0.06 (**moderate effect**), η*^2^* ≈ 0.14 (**large effect**)  ^a^ Kruskal Wallis test statistics due to assumption violation, ^b^ Based on square root transformed values, ^c^ Test statistic based on winsorized data | | | | | | | | | | |

| Table S4: Group differences in sleepiness (one-way ANOVA and linear mixed model) | | | | | | | | | | |
| --- | --- | --- | --- | --- | --- | --- | --- | --- | --- | --- |
| **Measure** | **Total *M*** | ***SD*** | **Suvorexant *M*** | ***SD*** | **Temazepam *M*** | ***SD*** | **Placebo *M*** | ***SD*** | ***p*** | ***η^2^*** |
| Average | 3.92 | 1.46 | 4.95 | 1.50 | 3.60 | 1.27 | 3.20 | 1.09 | .01** | **0.27** |
| Trial 1 | 4.33 | 1.81 | 5.20 | 2.04 | 4.20 | 1.69 | 3.60 | 1.43 | .16^a^ | 0.06 |
| Trial 2 | 3.67 | 1.56 | 4.40 | 1.65 | 3.80 | 1.69 | 2.80 | 0.92 | .06 | **0.18** |
| Trial 3 | 3.53 | 1.70 | 4.50 | 1.90 | 3.30 | 1.64 | 2.80 | 1.14 | .07 | **0.18** |
| Trial 4 | 3.67 | 1.69 | 4.90 | 1.79 | 3.30 | 1.49 | 2.80 | 1.03 | .02*^b^ | **0.26** |
| Trial 5 | 3.50 | 1.57 | 4.70 | 1.64 | 3.00 | 1.16 | 2.80 | 1.23 | .001** | **0.30** |
| *Notes*: n=30. KSS: Karolinska Sleepiness Scale (1: Extremely alert, 5: neither alert nor sleepy, 10: Extremely sleepy, can’t keep awake)  η*^2^* ≈ 0.01 (small effect), η*^2^* ≈ 0.06 (**moderate effect**), η*^2^* ≈ 0.14 (**large effect**)  ** p* ≤ .05, *** p* ≤ .01  ^a^ Kruskal Wallis test statistics due to nonnormality, ^b^ Test statistic based on sqrt transformed data | | | | | | | | | | |

| Table S5: KSS model summary (Type III ANOVA) | | | | | | |
| --- | --- | --- | --- | --- | --- | --- |
| fixed effects | Sum of Squares | Error Sum Sq | *df_Num_* | *df_Den_* | *F* | *p* |
| Drug | 82.84 | 211.62 | 2 | 27 | 5.28 | .01* |
| KSS trial | 13.89 | 104.08 | 4 | 108 | 3.60 | .01** |
| Interaction | 4.43 | 104.08 | 8 | 108 | 0.57 | .80 |
| *Notes*: n=30. KSS: Karolinska Sleepiness Scale (1: Extremely alert, 5: neither alert nor sleepy, 10: Extremely sleepy, can’t keep awake), *df_Num_*: degrees of freedom numerator, *df_Den_*: degrees of freedom denominator.  P-values based on Greenhouse-Geisser and Huyn-Feldt correction due to departure from sphericity.  ** p* ≤ .05, *** p* ≤ .01 | | | | | | |

| Table S6: Group differences in PVT lapses (one-way ANOVA) | | | | | | | | | | |
| --- | --- | --- | --- | --- | --- | --- | --- | --- | --- | --- |
| **Measure** | **Total *M*** | ***SD*** | **Suvorexant *M*** | ***SD*** | **Temazepam *M*** | ***SD*** | **Placebo *M*** | ***SD*** | ***p*** | ***η^2^*** |
| Average | 5.35 | 5.08 | 6.74 | 5.84 | 5.42 | 6.01 | 3.88 | 2.67 | .47 | **0.06** |
| Trial 1 | 5.75 | 7.40 | 7.56 | 9.14 | 6.67 | 8.94 | 3.30 | 2.87 | .44 | **0.06** |
| Trial 2 | 4.54 | 5.55 | 5.89 | 8.10 | 4.00 | 5.01 | 3.78 | 2.64 | .10^a^ | **0.06** |
| Trial 3 | 5.89 | 6.59 | 6.22 | 7.53 | 6.5 | 8.25 | 5.00 | 4.27 | .99 | 0.00 |
| Trial 4 | 5.59 | 4.73 | 6.30 | 4.79 | 5.50 | 5.21 | 4.89 | 4.68 | .89 | 0.00 |
| Trial 5 | 5.38 | 5.56 | 7.67 | 6.08 | 4.90 | 6.79 | 3.80 | 2.97 | .24 | **0.10** |
| *Notes*: n=30. PVT: psychomotor vigilance test lapses (reaction time >500ms). Test statistic based on sqrt transformed data unless nonparametric test is used.  η*^2^* ≈ 0.01 (small effect), η*^2^* ≈ 0.06 (**moderate effect**), η*^2^* ≈ 0.14 (**large effect**)  ^a^ Kruskal Wallis test statistics due to assumption violation | | | | | | | | | | |

| Table S7: PVT lapses model summary (Type III ANOVA) | | | | | | |
| --- | --- | --- | --- | --- | --- | --- |
| fixed effects | Sum of Squares | Error Sum Sq | *df_Num_* | *df_Den_* | *F* | *p* |
| Drug | 3 | 173.12 | 2 | 21 | 0.19 | .83 |
| PVT trial | 1 | 27.71 | 4 | 84 | 1.00 | .41 |
| Interaction | 3 | 27.71 | 8 | 84 | 1.03 | .42 |
| *Notes*: n=30. PVT: Psychomotor vigilance test lapses (reaction time >500ms), *df_Num_*: degrees of freedom numerator, *df_Den_*: degrees of freedom denominator | | | | | | |
